# Data on the clinical, analytical, and laboratory factors associated with negative anion gaps at an academic medical center

**DOI:** 10.1016/j.dib.2022.108357

**Published:** 2022-06-06

**Authors:** Joseph M. Laakman, Zachary J. Fleishhacker, Matthew D. Krasowski

**Affiliations:** a Department of Pathology, University of Iowa Hospitals and Clinics, Iowa City, IA 52242, USA; b Carver College of Medicine, University of Iowa, Iowa City, IA, USA

**Keywords:** Acid-base equilibrium, Anion gap, Clinical laboratory services, Hypoalbuminemia, paraproteinemias, Respiratory acidosis

## Abstract

The anion gap is a calculated parameter derived from the difference between the major plasma cations and anions in serum/plasma or whole blood, with a widely used simple equation utilizing concentrations of sodium, chloride, and bicarbonate. While there is extensive literature on the clinical significance and causes of elevated anion gaps, there is comparatively less data on low anion gaps. Occasionally, anion gap calculations result in a negative number (-1 or less). From the published literature, causes of these 'negative anion gaps' include laboratory error, specimen contamination or interference, hypoalbuminemia, extreme hyperkalemia, bromism, and paraproteins from multiple myeloma or similar pathologic processes. The data in this article present results from retrospective review of clinical chemistry and blood gas analysis testing at an academic medical center. The data include electrolyte concentrations and anion gap values derived from a total of 2,948,574 specimens (2,841,863 serum/plasma specimens analyzed on Roche Diagnostics clinical chemistry analyzers, 93,987 whole blood specimens analyzed on Radiometer blood gas analyzers, and 12,724 whole blood specimens on point-of-care chemistry devices) from 371,925 unique patients, clinical area where testing was ordered (for serum/plasma samples), sex, and age. For serum/plasma specimens with a negative anion gap, the data additionally include information from detailed chart review of possible factors and disease conditions contributing to the negative anion gap, pattern of electrolyte abnormalities, presence or absence of hypoalbuminemia, and corrected anion gap (if hypoalbuminemia is present).

## Specifications Table

**Table:** 

Subject	Medicine and Dentistry
Specific subject area	Pathology and Medical Technology
Type of data	Figure Tables Supplemental files
How data were acquired	Retrospective chart and data review from laboratory analysis performed at an academic medical center central clinical laboratory were obtained via tools within the electronic medical record.
Data format	Raw and Analyzed
Parameters for data collection	Retrospective data on clinical chemistry and blood gas analyzer testing that included sodium concentration [Na^+^], bicarbonate concentration [HCO_3_^−^], and chloride concentration [Cl^−^] in either serum/plasma (clinical chemistry analyzers) or whole blood (blood gas analyzers) were obtained from the electronic medical record (Epic, Inc.) covering the time period from May 1, 2009 through August 18, 2021. Detailed chart review was performed for all cases where calculated anion gap in serum/plasma yielded a negative number (-1 or less; 'negative anion gap'). Calculated anion gap used the equation Anion Gap = [Na^+^] – [HCO_3_^−^] – [Cl^−^], with the electrolyte concentrations in mEq/L. The project had approval from the University of Iowa Institutional Review Board (protocols # 202108527 and 202010420).
Description of data collection	There were a total of 2,841,863 serum/plasma specimens from 365,541 unique patients (187,982 female and 177,559 male), 93,987 whole blood specimens from 15,715 unique patients (6,861 female and 8,854 male), and 12,724 whole blood specimen from 5,370 unique patients (3,264 female and 2,106 male) analyzed on point-of-care chemistry analyzers from the retrospective time period. Accounting for overlap for patients who had both serum/plasma and whole blood specimens analyzed in the retrospective timeframe, there were a total of 2,948,574 specimens from 371,925 unique patients (191,452 female and 180,473 male). Detailed chart review was performed on all serum/plasma specimens that yielded a negative anion gap. For serum/plasma specimens, anion gap could be calculated from either panels of testing (e.g., the 8-test Basic Metabolic Panel) or from discrete (individual) orders for the 3 electrolytes on the same specimen. For serum/plasma specimens, the data collection contained results of the following: order description from which the anion gap derived (e.g., Basic Metabolic Panel or blood gas analyzer electrolytes), clinical location from which specimen was obtained [emergency department, outpatient clinic including phlebotomy stations, intensive care unit (ICU), or non-ICU inpatient unit], patient age in years at time of specimen collection, sex of patient as in the electronic medical record, [Na^+^] in mEq/L, [HCO_3_^−^] in mEq/L, [Cl^−^] in mEq/L, and calculated anion gap. For whole blood specimens, clinical location at time of specimen location was unknown; otherwise, the other elements described above for serum/plasma specimens were available. For those serum/plasma specimens with a negative anion gap, detailed chart review provided the additional data elements: identification of a recognized cause of
	low anion gap in the patient, other contributory disease processes that may have influenced anion gap, additional comments from chart review, classification of the electrolyte abnormalities, presence or absence of hypoalbuminemia, and corrected anion gap (if hypoalbuminemia was present).
Data source location	University of Iowa Hospitals and Clinics, Iowa City, Iowa, United States of America
Data accessibility	Four tables and two figures are included within the paper. 9 Supplementary files are deposited in Mendeley: Data identification number: 10.17632/jm7287z8dr.3 Direct URL to data: https://data.mendeley.com/datasets/jm7287z8dr/3

## Value of the Data

• The data provided are of value as negative anion gap may convey important pathophysiologic information or suggest the presence of an error affecting the specimen results.
• Clinicians, other researchers, or personnel in clinical laboratories might find this data useful as a reference for comparison.
• The data reinforce prior studies that specimen issues or laboratory error account for many negative anion gaps.
• The data include point-of-care clinical chemistry data, for which little prior published data exist.
• The data provide information for total of 2,948,574 specimens from 371,925 unique patients.

## 1 Data

Anion gap is a value calculated by determining the difference between major plasma cations (sodium, Na^+^; sometimes additionally potassium, K+) and anions (e.g., bicarbonate, HCO_3_^−^; chloride, Cl^−^), with “normal” levels (reference range) of approximately 8 to 16 mEq/L depending on analytical methodology, specimen type, and patient population [1], [2], [3], [4], [5], [6]. There is very limited data on anion gap reference ranges in children [7]. We did not find any publications on reference range or distribution of anion gap in point-of-care analyzers except for blood gas analyzers. Although many anion gap equations of various complexities have been proposed in the literature, a widely used simple equation is the following: Anion Gap = [Na^+^] – [HCO_3_^−^] – [Cl^−^], with concentrations in mEq/L [2], [3], [4]. Anion gap may be determined manually or calculated automatically and documented in the electronic medical record.

Elevations in anion gap are classically described in a variety of different clinical disease states, including but not limited to drug toxicities (e.g., ethylene glycol, methanol, salicylates), ketoacidosis, and lactic acidosis [2], [3], [4],8]. Comparatively less is understood about the etiology of anion gaps below the reference range, including 'negative anion gaps’ with values of -1 or lower [9], [10], [11], [12]. Certain pathologic states, drugs, and laboratory abnormalities have been associated with low anion gap measurements [1,^6^,^8^,13]. These include hypoalbuminemia [8,[14], [15], [16], chronic respiratory acidosis with compensatory metabolic alkalosis [17], hypercalcemia [18], hypermagnesemia [19], marked hyperkalemia [9], polyclonal gammopathy [20], presence of paraproteins (e.g., multiple myeloma) [21,22], pseudohyponatremia (due to factors such as hypertriglyceridemia, severe hypercholesterolemia, or hyperproteinemia) [23], pseudohyperbicarbonemia (such as due to ketoacidosis or monoclonal proteins) [9], bromism (intoxication with bromides) [24], iodine administration [10], lithium toxicity [19,25], thiosulfate [26], salicylate poisoning [9,12], and administration of the antimicrobial polymyxin B [27]. Previous studies have also identified pre-analytical specimen issues (e.g., contaminated specimens), analytical errors, and post-analytical errors (e.g., data entry mistake into the medical record) as a relatively frequent cause of low anion gaps [1,^6^,28]. Errors that could result in low anion gaps would include those that result in some combination of erroneously low [Na^+^], high [HCO_3_^−^], and/or high [Cl^−^] [9]. In the present study, we grouped these type of errors as “spurious events”.

This retrospective study includes data on a total of 2,841,863 serum/plasma specimens from 365,541 unique patients (187,982 female and 177,559 male) over a 12-year period at an academic medical center. We performed detailed chart review on all serum/plasma specimens that yielded a negative anion gap, encompassing data from 133 serum/plasma specimens from 116 unique patients (52 female, 64 male). The detailed chart review assessed whether factors associated with negative anion gaps in the published literature were present for the specimens with a negative anion gap. We also identified additional factors that may have contributed to the low anion gap.

**Fig. 1:**
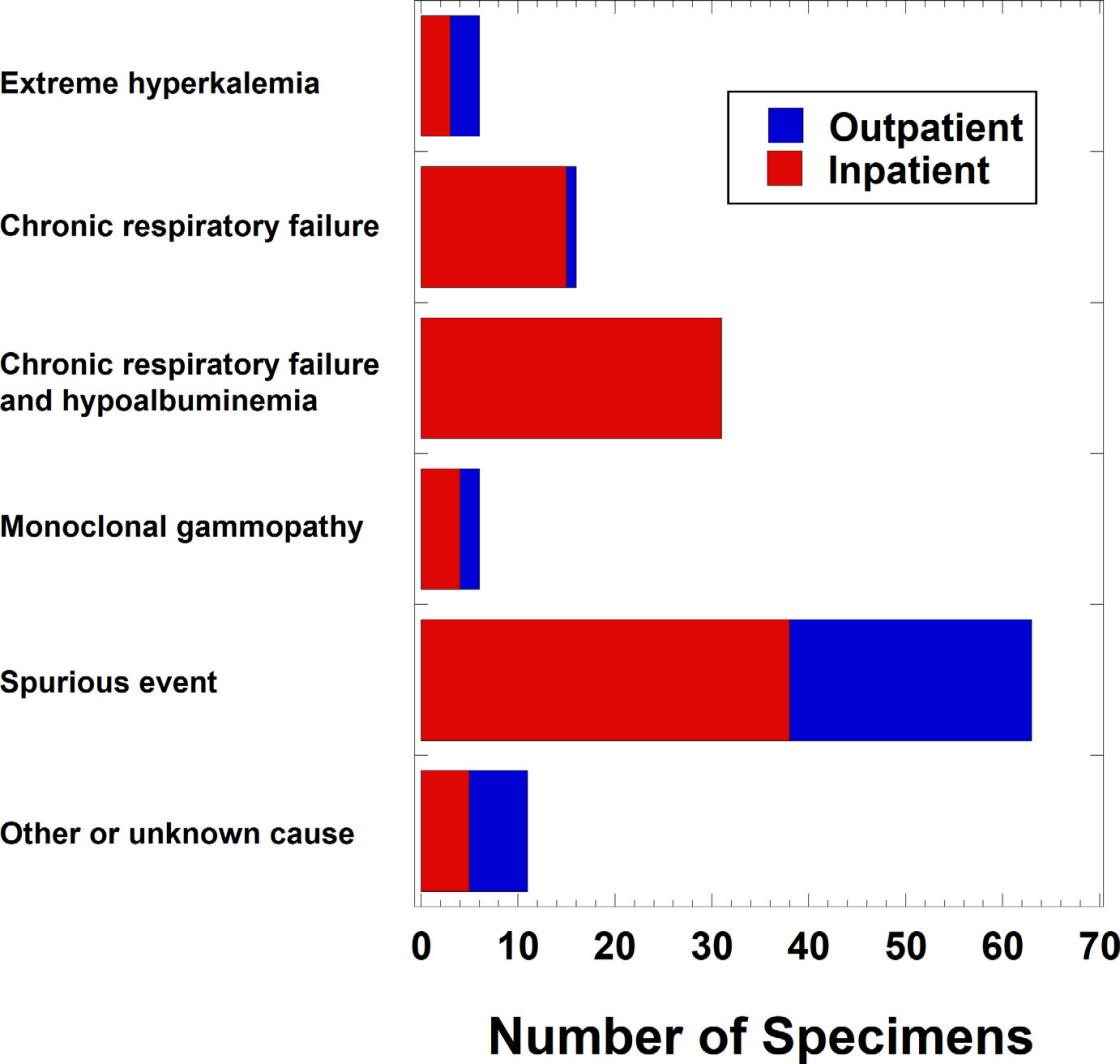
Breakdown of suspected causes of negative anion gaps in serum/plasma samples. The data is sorted by outpatient (including emergency department) and inpatient (including intensive care units). The underlying primary data is in Supplementary file 2.

Negative anion gaps in serum/plasma specimens were uncommon overall (133 of 2,841,863 specimens, 0.0047%), occurring in only 4 of 347,554 (0.0012%) of emergency department orders, 25 of 420,545 (0.0059%) of ICU orders, 71 of 1,114,191 (0.0064%) of non-ICU inpatient orders, and 33 of 959,573 (0.0034%) of outpatient orders. Table 1 shows a breakdown of suspected factors associated with serum/plasma specimens that had a negative anion gap, with spurious events, chronic respiratory acidosis with compensatory metabolic alkalosis, hypoalbuminemia, and paraproteins identified as the most common suspected causes. Hypoalbuminemia was present in 39 of the 133 (29.3%) specimens, including 31 of the 47 (66.0%) specimens associated with patients with chronic respiratory acidosis and compensatory metabolic alkalosis. In 34 of 133 specimens (25.6%) with a negative anion gap, calculation of a Corrected Anion Gap (calculated as Anion gap + (2.5 x ([Normal Albumin] – [Measured Albumin])), with [Albumin] measured in concentrations of g/dL) resulted in a value of 0 or greater. Fig. 1 shows a breakdown of suspected negative anion gap causes in outpatients (including patients seen at emergency department) and inpatients (including those in ICUs). The occurrence of a specimen with negative anion gap in a patient with chronic respiratory failure was seen almost entirely in inpatients and not outpatients. In contrast, spurious events, extreme hyperkalemia, and monoclonal gammopathies associated with negative anion gaps occurred in both inpatients and outpatients.

**Table 1:** Suspected causes of negative anion gap run on automated clinical chemistry analyzers.

Likely reason for negative anion gap^1^	Number of unique patients with one or more specimens in the category^2^	Number of samples^2^
Spurious event (e.g., laboratory error, contaminated specimen)	63	63
Chronic respiratory acidosis with compensatory metabolic alkalosis	36	47
Hypoalbuminemia	29	38
Hypercalcemia	0	0
Hypermagnesemia	0	0
Polymyxin B	0	0
Polyclonal gammopathy	0	0
Monoclonal proteins	6	6
Marked hyperkalemia	1	6
Lithium toxicity	0	0
Pseudohyponatremia – hypertriglyceridemia	0	0
Pseudohyponatremia – marked hypercholesterolemia	0	0
Pseudohyponatremia – hyperproteinemia	0	0
Pseudohyperbicarbonemia – ketoacidosis	0	0
Bromism	0	0
Iodine	0	0
Thiosulfate	0	0
Salicylate poisoning	0	0

1 Items in this column are those recognized in the published literature as associated with negative anion gaps.

2 Attribution of cause determined by detailed chart review of laboratory studies and clinical documentation. Spurious events were those with at least two of the following: (a) no other explanation likely given clinical history, (b) result inconsistent with baseline and/or follow-up laboratory studies, (c) clinical documentation attributing the result to “laboratory error” or similar language, or (d) cluster of similar suspicious results on same day.

Table 2 summarizes other medical conditions or situations associated with the serum/plasma specimens with a negative anion gap. This table contains factors not included in Table 1. Five specimens from 3 unique patients were obtained shortly before death in ICU patients who had received interventions immediately before expiring. All 3 unique patients in this situation had extreme electrolyte abnormalities such as hyperchloremia of 195 mEq/L in one patient. Table 3 summarizes the patterns of electrolyte abnormalities that contributed to a negative anion gap in serum/plasma specimens. The two most common patterns were: isolated elevation of HCO_3_^−^ (46 of 133 or 34.6% of specimens with negative anion gap; this was typical of patients with chronic respiratory acidosis with compensatory metabolic alkalosis) and isolated decrease of [Na^+^] (27 of 133, 20.3%; common in spurious events such as suspected laboratory error). The least common pattern was a combination of low [Na^+^], high [HCO_3_^−^], and high [Cl^−^] (1 of 133, 0.8%)

**Table 2:** Medical conditions or situations associated with specimens with a negative anion gap run on automated clinical chemistry analyzers.

Factor^1^	Number of unique patients	Number of samples
Perimortem^2^	3	3
Other^3^	5	5
Unknown	3	3

1 These may or may not have contributed to the low anion gap but were the main presentation at time of specimen collection.

2 Three cases were laboratory values obtained in patients in intensive care units during interventions immediately before death. All three cases had extreme hypernatremia (165 mEq/L or higher) and two had extreme hyperchloremia (134 mEq/L or higher). These were notably different from prior laboratory studies.

3 There was one case of each of the following: congenital heart defect (Tetralogy of Fallot) inborn error of metabolism (fatty acid oxidation pathway), severe nausea and vomiting, prolonged diarrhea, and tumor lysis syndrome (leukemia).

**Table 3:** Breakdown of electrolyte abnormalities in specimens with a negative anion gap run on automated clinical chemistry analyzers.

Specimen category				

Na^+^ low^1^	Cl^-^ high^2^	HCO_3_^−^ high^2^	Unique patients with one or more specimens in the category^3^	Specimens in the category^3^ (number / %)
X			18	27 (20.3%)
	X		10	16 (12.0%)
		X	24	46 (34.6%)
X	X		14	17 (12.8%)
X		X	8	20 (15.0%)
	X	X	5	6 (4.5%)
X	X	X	1	1 (0.8%)

1 Indicates whether the Na^+^ was below lower limit of reference range for patient age at time of specimen collection.

2 Indicates whether the Cl^-^ or HCO_3_^−^ was above upper limit of reference for patient age at time of specimen collection.

3 Note that some patients had multiple specimens with different categories so that sum of results exceeds number of unique patients.

The retrospective study also included data from 93,987 whole blood specimens from 15,715 unique patients (6,861 female and 8,854 male) that were analyzed on blood gas analyzers. A total of 62,056 specimens (66.0% of the 93,987 specimens analyzed) were from 3,649 infants who were less than 1 years old at the time of specimen collection. This infant test volume reflects a busy neonatal ICU within the medical center that serves as a regional referral center for complex obstetric and neonatal issues. Due to informatics challenges in retrieving this data after a switch in the laboratory information system in August 2014, the retrospective timeframe is limited to May 1, 2009 to August 2, 2014, when complete data could be retrieved. In addition, patient location at time of order could not be retrieved through this search.

Table 4 shows a summary of the electrolyte abnormalities in whole blood specimens with a negative anion gap measured on the blood gas analyzer. Negative anion gaps were much more common in the blood gas analyzer dataset than in the serum/plasma dataset described above. Overall, 886 out of 93,987 (0.94%) whole blood specimens had a negative anion gap on the blood gas analytes, but this was strongly influenced by a higher percentage of negative anion gaps in the infant population less than 1 year old (829 of 62,056 specimens, 1.3%) compared to only 57 of 31,931 specimens (0.18%) in all other ages. To our knowledge, there is no detailed published investigation of negative anion gaps in the newborn/infant population, including premature infants that may be hospitalized in neonatal intensive care units. The most common patterns overall in the blood gas analyzer data were low Na^+^ with high [Cl^−^] (299 of 886 specimens, 33.7%) and [Cl^−^] and [HCO_3_^−^] both high (188 of 886 specimens, 21.2%).

**Table 4:** Breakdown of electrolyte abnormalities in specimens with a negative anion gap run on blood gas analyzers.

Specimen category						
						
Na^+^ low^1^	Cl^-^ high^2^	HCO_3_^−^ high^2^	Unique patients with one or more specimens in the category (all patients)^3^	Specimens in the category (all patients)^3^ (number / %)	Unique patients with one or more specimens in the category (infants only)^3^	Specimens in the category (infants only)^3^ (number / %)
X			38	48 (5.4%)	25	34 (4.2%)
	X		64	76 (8.6%)	46	57 (7.1%)
		X	107	122 (13.8%)	81	187 (23.3%)
X	X		153	299 (33.7%)	84	95 (11.8%)
X		X	93	138 (15.6%)	142	288 (35.9%)
	X	X	82	188 (21.2%)	87	130 (16.2%)
X	X	X	15	15 (1.7%)	12	12 (1.5%)

1 Indicates whether the Na^+^ was below lower limit of reference range for patient age at time of specimen collection.

2 Indicates whether the Cl^-^ or HCO_3_^−^ was above upper limit of reference for patient age at time of specimen collection.

3 Note that some patients had multiple specimens with different categories so that sum of results exceeds number of unique patients.

**Fig. 2:**
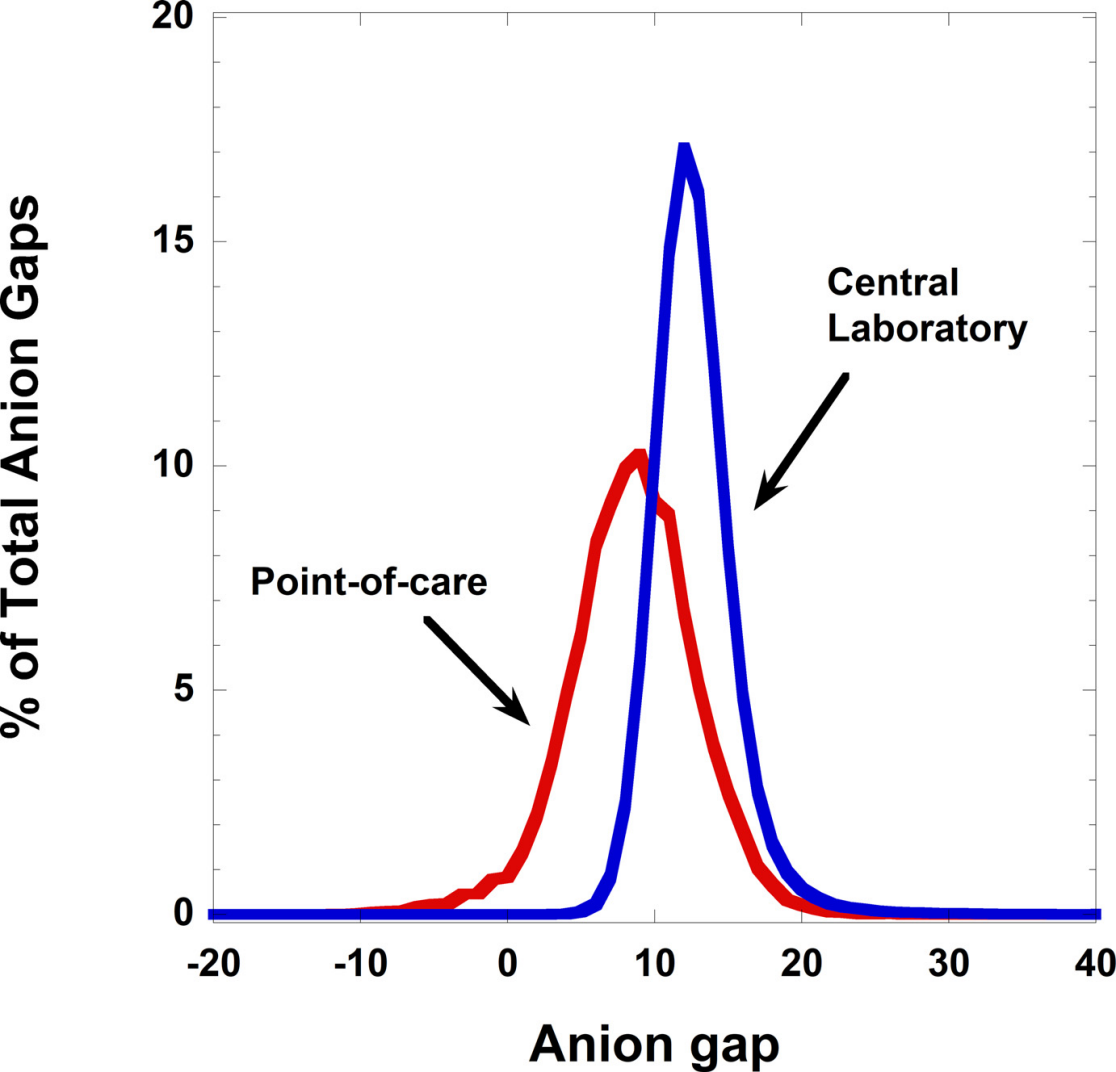
Anion gap distributions from point-of-care and central laboratory testing. The underlying primary data is in Supplementary file 9.

We also analyzed data from point-of-care chemistry analyzers (Abaxis Piccolo) from four outpatient locations (two Urgent Care sites, one pediatric clinic, and one hematology/oncology clinic) that used these devices for near point-of-care testing. The retrospective timeframe for the point-of-care analysis was July 1, 2017 through October 28, 2020. This data is a subset of a detailed analysis of electronic interfacing of point-of-care analyzers published in a separate study [29]. In this same retrospective timeframe, we also analyzed central laboratory data for all outpatient clinic sites in our health system that were analyzed in the central medical center laboratories by Roche Diagnostics methodology (this is a subset of the serum/plasma data discussed above). This central laboratory data serves as an outpatient comparison for the same timeframe. Negative anion gaps were much more common in the point-of-care dataset, with 22 of 1,585 (1.39%) specimens for basic metabolic panels and 309 of 11,139 (2.77%) specimens for comprehensive metabolic panels on the Piccolo having a negative anion gap. In contrast, the rate for all outpatient specimens analyzed in the central laboratory was < 0.01%. Fig. 2 shows the distribution of anion gaps for the point-of-care compared to central laboratory data, with the distribution for point-of-care trending towards lower values.

There are multiple possibilities for why point-of-care anion gaps trend lower than the central laboratory. The first possibility is that one or more analytes in the anion gap calculation are systematically biased by the point-of-care analytical methodology relative to the central laboratory analytical methodology in the direction of lower [Na^+^], higher [HCO_3_^−^], and/or higher [Cl^−^]. In a calculation involving three analytes, a combination of systematic biases could shift anion gaps to lower values. The second possibility is that there are errors or interferences that impact the point-of-care measurements to a higher degree than central laboratory measurement. [30,31] This may account for some sporadic low anion gaps in point-of-care samples. The third possibility is that the sample type in point-of-care (whole blood) relative to central laboratory (plasma/serum) influences the measurement of [Na^+^], [HCO_3_^−^], and/or [Cl^−^]. The use of different sample types also makes direct comparison of patient samples between methods more challenging. Lastly, checks for sample quality (e.g., hemolysis, icterus, and lipemia, bubbles) are more robust in central laboratory methods using plasma/serum than point-of-care methods utilizing whole blood [30], [31], [32]. Hemolysis, in particular, is difficult to detect in whole blood samples and is not obvious visually to the operator.

The raw data for the study are included in Supplementary files 1-9.

• Supplementary file 1: Breakdown of the data sources in the study which are included in Supplemental files 2-9. Specific data fields include: data source, corresponding supplemental file that has the raw data, start date of retrospective review, end date of retrospective review, total number of specimens, number of unique patients, number of females (using sex as recorded in electronic medical record), number of males, average patient age in years, median patient age in years, minimum patient age in years, maximum patient age in years, number of specimens with negative anion gap (-1 or less), percent of specimens with negative anion gap, number of specimens that were obtained in emergency department, number of specimens that were obtained in intensive care units, number of specimens that were obtained in other inpatient units, number of specimens that were obtained in outpatient setting, percent of total specimens that were obtained in the emergency department, percent of total specimens that were obtained in the intensive care units, percent of total specimens that were obtained in other inpatient units, and percent of total specimens that were obtained in the outpatient setting. Patient location data was not available for the subset of data obtained from blood gas analyzers due to complexities with the data source and retrieval. Note that there is some overlap in patient populations in some of the files.
• Supplementary file 2: Data from 133 serum/plasma specimens from 116 unique patients (52 female, 64 male) that had a negative anion gap (-1 or lower).
• Supplementary file 3: Data for 886 whole blood specimens from 387 unique patients (166 female, 221 male) that had a negative anion gap (-1 or lower).
• Supplementary file 4: Data for 776,504 specimens from 167,763 unique patients who had the Basic Metabolic Panel (sodium, potassium, chloride, bicarbonate, creatinine, blood urea nitrogen, glucose, total calcium) ordered which yielded an anion gap or 11 or lower. Overlap with the data in Supplemental file 5 yields a total of 1,581,143 specimens from 252,310 unique patients who had the Basic Metabolic Panel performed.
• Supplementary file 5: Data for 804,639 specimens from 184,466 unique patients who had the Basic Metabolic Panel (sodium, potassium, chloride, bicarbonate, creatinine, blood urea nitrogen, glucose, total calcium) ordered which yielded an anion gap or 12 or higher.
• Supplementary file 6: Data for 809,704 specimens from 183,976 unique patients who had discrete (individual orders) for sodium, chloride, and bicarbonate on the same specimen.
• Supplementary file 7: Data for 451,016 specimens from 121,862 unique patients who had orders for Electrolyte Panel (sodium, potassium, chloride, bicarbonate), Chemistry 6 panel (sodium, potassium, chloride, bicarbonate, creatinine, blood urea nitrogen), Chemistry 7 panel (sodium, potassium, chloride, bicarbonate, creatinine, blood urea nitrogen, glucose), or Comprehensive Metabolic Panel (sodium, potassium, chloride, bicarbonate, creatinine, blood urea nitrogen, glucose, total calcium, total bilirubin, alanine aminotransferase, aspartate aminotransferase, albumin, alkaline phosphatase, total protein).
• Supplementary file 8: Data for 93,987 specimens from 15,715 unique patients who had blood gas analysis performed on whole blood specimens where concentrations for sodium, chloride, and bicarbonate were all determined.
• Supplementary file 9: Data for 361,199 specimens from 127,230 unique patients who had basic metabolic panels performed on either Piccolo Express (1,585 specimens) or in the central laboratory (185,277 specimens) or comprehensive metabolic panels performed on either Piccolo Express (11,139 specimens) or in the central laboratory (163,198 specimens).

## 2 Experimental Design, Materials and Methods

### 2.1 Data source

All retrospective data in the study related to serum/plasma and point-of-care analysis was obtained via Epic Reporting Workbench, which allows for extraction of data from the electronic medical record [33]. Although the overall retrospective analysis period was May 1, 2009 through August 18, 2021 for the serum/plasma specimens, various chemistry test panels were introduced as orderable panels in the electronic order entry system at various times starting with January 11, 2010 for the Basic Metabolic Panel. Supplemental file 1 lists the start date for when the other panels were introduced in the electronic order entry system. The blood gas analyzer data was retrieved from the laboratory information system used in our clinical laboratories until October 2014 (Cerner Classic, Kansas City, MO, USA). Patient location at the time of test ordering was unavailable for the blood gas analyzer data. The point-of-care chemistry data was from a larger dataset analyzed in a separate published study [29]. Data extracted from Epic Reporting Workbench or Cerner Classic involved only patients who received medical care at the University of Iowa Hospitals and Clinics (Iowa City, Iowa, United States). Detailed chart review was performed for all patients who had a negative anion gap on a serum/plasma specimen.

### 2.2 Calculations and definitions

Calculated anion gap used the equation Anion Gap = [Na^+^] – [HCO_3_^−^] – [Cl^−^], with the electrolyte concentrations in mEq/L. Corrected anion gap used the following equation: Corrected Anion Gap = Anion gap + (2.5 x ([Normal Albumin] – [Measured Albumin])), with [Albumin] measured in concentrations of g/dL [16]. For assessment whether a spurious event such as laboratory error or specimen contamination was likely to have caused the negative anion gap, the specimen must have been associated with at least 2 of the following: (a) no other explanation likely given clinical history, (b) result inconsistent with baseline and/or follow-up laboratory studies, (c) clinical documentation attributing the result to “laboratory error” or similar language, or (d) cluster of similar suspicious results on same day. For the purposes of the current study, the following were considered recognized causes of low anion gap from the available literature: spurious event (such as laboratory error or contaminated specimen), chronic respiratory acidosis with compensatory metabolic alkalosis, hypoalbuminemia, hypercalcemia, hypermagnesemia, administration of polymyxin B, polyclonal gammopathy, presence of monoclonal proteins (e.g., multiple myeloma), marked hyperkalemia, lithium toxicity, pseudohyponatremia due to hypertriglyceridemia, pseudohyponatremia due to marked hypercholesterolemia, pseudohyponatremia due to hyperproteinemia, pseudohyperbicarbonemia due to ketoacidosis, bromism, iodine administration, thiosulfate, or salicylate poisoning. Contributory disease processes that may affect anion gap included the following: multiple myeloma and other monoclonal gammopathies, acute myeloid leukemia, and chronic obstructive pulmonary disease. We also noted some specimens with negative anion gap that were collected just prior to patient expiring from critical illness. In that setting, unusual electrolyte patterns may be due to aggressive intervention measures and/or derangements related to a disease process resulting in death.

### 2.3 Analytical methology

Up to early 2013, electrolyte analysis on serum/plasma specimens was performed on Roche Diagnostics (Indianapolis, IN, USA) Modular P analyzers. This instrumentation was replaced in 2013 with cobas 8000 system c702 analyzers. Blood gas analysis, including electrolyte concentrations measured in the whole blood, was performed on Radiometer (Copenhagen, Denmark) ABL835 analyzers in the retrospective timeframe of this report. The point-of-care chemistry analysis was performed on the Abaxis Piccolo Xpress using the basic metabolic panel and comprehensive metabolic panel cartridges [29].

## Ethics Statement

The analyses had approval by the University of Iowa Institutional Review Board (protocols # 2020104420 and 202108527) as a retrospective project with waiver of informed consent. The research was carried out in accordance with The Code of Ethics of the World Medical Association (Declaration of Helsinki).

## CRediT authorship contribution statement

**Joseph M. Laakman:** Formal analysis, Writing – review & editing. **Zachary J. Fleishhacker:** Conceptualization, Writing – original draft, Writing – review & editing, Formal analysis. **Matthew D. Krasowski:** Formal analysis, Conceptualization, Writing – original draft, Writing – review & editing, Methodology, Supervision.

## Declaration of Competing Interest

The authors declare that they have no known competing financial interests or personal relationships that could have appeared to influence the work reported in this paper.

## Data Availability

Negative Anion Gaps (Original data) (Mendeley Data).
